# Adenoma of the Ampulla of Vater: A Genetic Condition?

**DOI:** 10.1155/1999/85232

**Published:** 1999-04

**Authors:** Francesco M. Serafini, Larry C. Carey

**Affiliations:** 1 Service of Gastrointestinal Surgery Tampa General Hospital Tampa Florida USA; 2 Department of Surgery University of South Florida Tampa Florida USA; 3 Department of Surgery University of South Florida 12901 Bruce B. Downs Boulevard MDC Box 16 Tampa Florida 33612 USA

## Abstract

The etiology of adenoma of the ampulla of Vater is not well understood. Previous authors reported the association of this neoplasm with polycystic kidney disease of two fraternal sisters. They concluded that these two conditions were somehow related. We describe a case of ampullary adenoma associated with polycystic kidney disease. This presentation raises again the question of a possible link between these two diseases.


*HPB Surgery*, 1999, Vol. 11, pp. 191–193
Reprints available directly from the publisher
Photocopying permitted by license only

© 1999 OPA (Overseas Publishers Association) N.V.
Published by license under
the Harwood Academic Publishers imprint,
part of The Gordon and Breach Publishing Group.
Printed in Malaysia.

*Case Report*

### Adenoma of the Ampulla of Vater: A Genetic Condition?

FRANCESCO M. SERAFINI [a] and LARRY C. CAREY [b,*]

[a] *Service of Gastrointestinal Surgery, Tampa General Hospital;*
[b] *Department of Surgery, University of South Florida, Tampa, Florida*

*(Received 15 December 1997)*

**The etiology of adenoma of the ampulla of Vater is not well understood. Previous authors reported the association of this neoplasm with polycystic kidney disease of two fraternal sisters. They concluded that these two conditions were somehow related. We describe a case of ampullary adenoma associated with polycystic kidney disease. This presentation raises again the question of a possible link between these two diseases.**

*Keywords:* Polycystic kidney disease, ampullary neoplasm, ERCP

### INTRODUCTION

Adenomas of the ampulla of Vater are rare causes of extrahepatic biliary obstruction. A thorough analysis and review of this condition is complicated by the rarity of the lesion and by the inconsistent terminology used to describe the neoplasm (papilloma, adenoma, ampulloma). The true incidence of such tumors is still not known. Ampullary adenomas are thought to be precancerous lesions and the malignant transformation of ampullary adenoma to adenocarcinoma has been described by several authors [1, 2].

The etiology of ampullary adenomas is not well understood. Genetic influences, however, may play an important role. Periampullary adenomas and adenomatous polyps of the upper gastrointestinal tract have frequently been associated with polyposis sydromes, as in familial adenomatous polyposis (FAP) and Gardner's syndrome [3, 4]. Further evidence of the association of these tumors and chromosome associated disorders was recently described by Norton who reported two biliary adenomas in two fraternal sisters both affected by adult polycystic kidney disease (APCKD) [5].

We describe an adenoma of the ampulla of Vater in a patient with adult polycystic kidney disease, and a strong family history of polycystic kidney disease. The purpose of this brief report is to emphasize the role of genetic influences in adenomas of the ampulla of Vater.

*Address for correspondence: Department of Surgery, University of South Florida, 12901 Bruce B. Downs Boulevard, MDC Box 16, Tampa, Florida 33612. Tel.: (813) 974-7094; Fax: (813) 974-2669.

### CASE REPORT

A 51 year old white female was referred with symptoms of cholangitis, including nausea, vomiting, fever and jaundice. She was known to have chronic renal failure related to adult polycystic kidney disease was anuric and being dialyzed three times weekly. Polycystic kidney disease was present throughout in her family (brother, grandfather), diagnosed with ultrasound and renal function testing. Initial laboratory testing demonstrated leukocyte count of 18,000/mm$^3$. total bilirubin of 4.6 mg/dl, hemoglobin of 8.6 g/dl, serum creatinine 6.2 mg/dl and BUN of 27 meq/L. A CT scan of the abdomen and pelvis demonstrated pronounced dilatation of the extrahepatic biliary tree with slight dilatation of Wirsung's duct. An endoscopic retrograde cholangiopancreatography showed obstruction at the papilla of Vater with pancreatic duct dilatation. Biopsy specimens obtained from the ampulla of Vater demonstrated the presence of a villous adenoma without dysplasia.

The patient underwent pylorus sparing Whipple's resection and definitive pathology confirmed the presence of a villous ampullary adenoma without malignant transformation. The postoperative course of this patient was uneventful and she was discharged in 10 days. Three months after discharge she underwent renal transplatation.

### DISCUSSION

Adenoma of the ampulla of Vater affect both sexes equally and usually present after the fifth decade of life. The incidence of this disease in postmortem series varies between 0.04% and 0.62%, with the majority being asymptomatic owing to their small size [5]. Yamaguchi reported 12 patients with ampullary adenomas. Only three had jaundice, the rest complained of vague abdominal pain or were asymptomatic [2]. Ampullary adenomas are thought to be precancerous lesions. The adenoma – carcinoma sequence, in adenomatous polyps of the colon – rectum, is also accepted for ampullary adenomas. Foci of carcinoma are frequently found in adenoma specimens and adenomatous tissue is often present in surgical specimens resected for vaterian cancer. Endoscopy is a useful tool in the diagnosis of ampullary neoplasms, even if endoscopic biopsy is unable to detect foci of carcinoma in ampullary adenomas. Blackman reported that endoscopic biopsy was inaccurate in excluding cancer in three of 20 patients who underwent surgical resection for ampullary neoplasm [6]. The ideal surgical management for these neoplasms remains controversial. Endscopic sphincterotomy and snare resection, transduodenal papillectomy, and Whipple's resection represent the most accepted alternatives of therapy. We think that pancreaticoduedenectomy represents the best approach in the management of such tumors, especially larger ones, because this modality offers better chances for pathological characterization, and can be curative in the presence of malignancy. Even though renal failure represents a comorbid factor for increased morbidity, we believe that Whipple's operations can be undertaken at tertiary referral centers with acceptable morbidity and mortality.

The etiology of adenomas of the ampulla of Vater is not known. There is speculation that environmental or local metabolic factors may be involved. In addition, evidence suggests that genetic influences may be implicated in the natural history of this disorder. Sanabria *et al.*, demonstrated that periampullary neoplasms in patients with FAP follow familial segregation patterns. By analyzing a large number of FAP families, they found that the occurrence of periampullary neoplasm in patients with FAP increased the likelihood that one of the other FAP patients from the same kindred will develop the same condition. They could not demonstrate a phenotype/genotype correlation between tendency to malignant transformation of ampullary adenomas and specific germline APC mutations [7]. Austin reported the occurrence of Vaterian adenocarcinoma in two male siblings, not affected by FAP, and presenting within two weeks of each other. Moreover, a third member from the same family, also not affected by FAP, died from periampullary cancer. Chromosome analysis of these patients demonstrated no morphologic abnormality with conventional G-banding, C-banding and karyotype analysis [8]. Norton *et al.*, recently described ampullary adenomas occuring in two fraternal sisters both affected by adult polycystic kidney disease (APCKD). Karyotype analysis was normal. They concluded that the likelihood of those two conditions to co-exist by chance was low [5] APCKD is an autosomal dominant inherited disease, even if the specific genetic defect responsible for this condition is not well known. Reeders *et al.*, using linkage analysis found that in 96% of patients the APCKD locus was closely linked (11 centimorgans) to the $\alpha$-globin locus on the short arm of chromosome 16 [9].

Our patient represents the second report in the literature where ampullary adenoma was associated to APCKD. Polycystic kidney disease was steadily present in her family. Ampullary adenoma had not been diagnosed in any other member of her family. However, such tumors are often asymptomatic because of their small size and they may be consequently overlooked Karyotype analysis was not available on this patient, because of the inhibitory influence of immunosuppressant therapy on peripheral leucocytes. The presentation of these two conditions together raises again the question of a possible genetic link. This hypothesis, at the present time, remains speculative.

### References

[1] Cattel, R. B. and Pyrtek, L. J. (1950). Premalignant lesions of the ampulla of Vater, *Surg. Gynecol. Obset.*, **90**, 21–30.

[2] Yamaguchi, K. and Enjoji, M. (1991). Adenoma of the ampulla of Vater: putative precancerous lesion, *Gutt.*, **32**, 1558–1516.

[3] Alexander, J. R., Andrews, J. M. and Buchi, K. N. (1989). High prevalence of adenomatous polyps of the duodenal papilla in familial adenomatous polyposis, *Dig. Dis. Sci.*, **34**, 167–170.

[4] Jones, T. R. and Nance, F. C. (1977). Periampullary malignancy in Gardner's syndrome, *Ann. Surg.*, **185**, 565–573.

[5] Norton, I. D., Pokorny, C. S., Painter, D. M., Johnson, J. R. and Perkins, K. W. (1995). Fraternal sisters with adult polycystic kidney disease and adenoma of the ampulla of Vater, *Gastroenterology*, **109**, 2007–2010.

[6] Blackman, E. and Nash, S. V. (1985). Diagnosis of duodenal and ampullary epithelial neoplasms by endoscopic biopsy: A clinicopathologic and Immunohistochemical study, *Human Pathology*, **16**, 901–910.

[7] Sanabria, J. R., Croxford, R., Berk, T. C., Cohen, Z., Bapat, B. V. and Gallinger, S. (1996). Familial segregation in the occurrence and severity of periampullary neoplasms in familial adenomatous polyps, *Am. J. Surg.*, **171**, 136–141.

[8] Austin, J. C., Organ, C. H., Williams, G. R. and Pitha, J. V. (1988). Vaterian cancer in siblings, *Ann. Surg.*, **207**, 655–661.

[9] Reeders, S. T., Breuning, M. H., Davies, K. E., Nicholls, R. D., Jarman, A. P., Higgs, D. R., Pearson, P. L. and Weatherall, D. J. (1985). A highly polymorphic DNA marker linked to adult polycystic kidney disease on chromosome 16, *Nature*, **317**, 542–544.

